# Factors associated with retention and adherence on Pre-Exposure Prophylaxis among men who have sex with men in Kigali, Rwanda

**DOI:** 10.1371/journal.pgph.0004063

**Published:** 2024-12-31

**Authors:** Sezi Mubezi, Samuel S. Malamba, Gallican N. Rwibasira, Jeanne Uwineza, Jean de Dieu Kayisinga, Eric Remera, Basile Ikuzo, Emah Ndengo, Nadege Umuhoza, Beata Sangwayire, Richard C. N. Mwesigwa, Caroline E. Stamatakis, Manasseh G. Wandera, Tom O. Oluoch, Eugenie Kayirangwa

**Affiliations:** 1 Health Program Unit, Society for Family Health (SFH), Kigali, Rwanda; 2 Division of Global HIV and TB, Global Health Center (GHC), US Centers for Disease Control and Prevention (CDC), Kigali, Rwanda; 3 Rwanda Biomedical Center (RBC), Ministry of Health, Kigali, Rwanda; 4 United States Agency for International Development (USAID), Kigali, Rwanda; University of Toronto, CANADA

## Abstract

Pre-Exposure Prophylaxis (PrEP) is recommended as an HIV prevention measure for men who have sex with men (MSM). We assessed factors associated with PrEP retention and adherence among MSM in Kigali, Rwanda. We undertook a retrospective cross-sectional study and used a questionnaire to obtain PrEP retention and adherence history from MSM enrolled in the key population (KP) program that attended scheduled follow-up clinics from four (4) health facilities between April 2021 to June 2021. Retention was defined as attending scheduled PrEP follow-up appointments and adherence as taking PrEP medication 95% or more of the time. We used multivariable cox proportion hazard regression to determine factors associated with 3-month retention and principal component analysis (PCA) to determine factors associated with self-reported adherence. Data were analyzed using STATA (version 16.0). We interviewed 439 MSM aged 18 years and above that were initiated on PrEP. Majority were employed (57%, n = 251), between ages 25–34 years (49%, n = 217), close to half completed primary level education (47%, n = 206), were involved in sex work (42%, n = 184), and over a half lived in household of 1–2 members (55%, n = 241). Ninety percent of the MSM respondents (n = 393) were retained on PrEP at 3 months and among those retained, 287 (73%) had good adherence. Multivariable cox regression revealed that MSM more likely to be retained on PrEP, were those that are sex workers (adjusted Hazard Ratio (aHR) = 4.139; 95% Confidence Interval (95%CI): 1.569, 10.921), had more than one (1) regular sexual partners (aHR = 3.949; 95%CI: 2.221, 7.022), lived in households of 3–5 members (aHR = 3.755; 95%CI: 1.706, 8.261), completed secondary school education (aHR = 2.154; 95%CI: 1.130, 4.108), and were circumcised (aHR = 2.218, 95%CI: 1.232, 3.993). Employed MSM had a 66% decreased likelihood to be retained on PrEP (aHR = 0.345; 95%CI: 0.168, 0.707). Similarly, MSM that used condoms consistently had an 85% decreased likelihood to be retained on PrEP (aHR = 0.149; 95%CI: 0.035, 0.632). Principal component regression analysis showed that the component with MSM with higher numbers of regular sexual partners had increased odds of adhering to PrEP (Crude Odds Ratio (cOR) = 1.32; 95%CI: 1.144, 1.530). The study highlighted that MSM using PrEP as the main method of HIV prevention were more likely to be retained and adherent to PrEP. There is need to emphasize PrEP use alongside other HIV prevention methods and targeted STI testing and treatment among PrEP users.

## Introduction

Pre-Exposure Prophylaxis (PrEP) is a prevention method in which people who do not have HIV but are at high risk of exposure to HIV, take a pill daily to reduce their risk of HIV infection. The pill contains two medicines that are also used to treat HIV: tenofovir disoproxil fumarate (TDF) and emtricitabine (FTC) commonly known as Truvada, or TDF and Lamivudine (3TC); and its efficacy in reducing HIV transmission was established in randomized controlled trials (RCTs) and open-label studies [1–3]. The successful implementation of PrEP has been demonstrated in diverse settings through demonstration projects, where it has demonstrated reduction in HIV transmission by up to 90% when taken correctly, compared with placebo; the actual efficacy achieved depending on adherence [4, 5]. PrEP may be used intermittently during periods of perceived HIV exposure, rather than continually and lifelong, as is the case with antiretroviral treatment (ART) [6].

PrEP has been demonstrated to prevent HIV in key populations (KPs) comprised of gay men and men who have sex with men (MSM), transgender (TG) people, and in persons who inject drugs (PWID). KPs are a populations of interest because of a heightened risk of HIV infection compared to the general populations due to biological, behavioral, clinical, and structural risk factors. PrEP holds potential to decrease new infections among KPs and is synergistic with efforts currently in place to achieve an end to the acquired immunodeficiency syndrome (AIDS) epidemic in sub-Saharan Africa (SSA) [7].

In 2021, it was reported that gay men and other MSM were 28 times more likely to acquire HIV than adult men in the general population globally [8]. Gay men and other MSM contributed to 41% and 6% of new HIV infections globally and in SSA respectively [8]. In Kigali, Rwanda, the HIV prevalence among MSM was reported as 10% compared to the general population HIV prevalence of 3% [9, 10]. This high burden of HIV among MSM in Rwanda highlights the need for combination HIV prevention for which PrEP is seen as an integral component [11].

On July 1^st^, 2018, Rwanda revised its national guidelines for prevention and management of HIV and STIs to include PrEP as an HIV prevention strategy, recommending PrEP for HIV-negative KPs who do not consistently use condoms, HIV negative people in sero-discordant relationships whose partners have not started ART or whose partners’ viral load is not suppressed (> 1000 viral copies/ml), and HIV negative sexually active adolescent girls and young women (AGYW) that are 18 years and above [12].

Among MSM enrolled in an HIV-1 vaccine preparedness open cohort study in Mombasa, Kenya, a PrEP retention rate of 65.3% was reported at the end of the 2-year study. In the same study, of the 76 participants that had blood samples taken to measure drug levels at 6 and 12 months, only 32 MSM (42%) had detectable TDF with only 11 of the 32 showing protective levels of the drug [13]. Another study from a large government hospital in coastal Kenya detected any TDF in blood samples of only 14.7% of MSM enrolled with none of them demonstrating protective levels of the drug at month 6 follow-up [14].

In Rwanda, same sex relations are decriminalized allowing MSM to receive a fully package of HIV prevention and treatment services as per the national guidelines for prevention and management of HIV. As per a 2021 study, it was estimated that Rwanda has an approximate population of about MSM of 18,100 aged 18 years and above [15]. Enrollments of KPs onto PrEP started in April 2018 but no comprehensive evaluation on the uptake, retention, and adherence of KP groups, MSM inclusive, had been conducted until the time of this study. As such, little was documented about PrEP retention and adherence levels, or the factors associated with PrEP retention and adherence. It ought to be noted that despite the scientific evidence supporting efficacy of event driven PrEP [16–18], the current Rwandan guidelines only provide for oral daily PrEP.

An interplay of factors like multiple sexual partnerships, use of alcohol and drugs, distance of households from nearest health center, the role of community and religious leaders in emphasizing the need for seeking healthcare from trained providers, the influence of peers, stigma, and discrimination might have a role in PrEP retention, and adherence. This study focused on enumerating the factors associated with PrEP retention and adherence among MSM in Kigali to provide insights and lessons for the national scale-up program.

## Materials and methods

### Study design and settings

We conducted a retrospective cross-sectional study where we cross-sectionally sampled the source population and then retrospectively assessed subjects’ histories of exposures over a specified period. We targeted MSM attending four (4) public health centers in the City of Kigali. These health centers are among the 21 supported facilities in the City of Kigali that are working with Society for Family Health (SFH) Rwanda to implement the KP program with funding from the Centers for Disease Control and Prevention (CDC). The KP program in Rwanda supports KPs, including MSM, mobilized by their peers to come to the health facilities to receive a comprehensive package of services for KPs that includes HIV Testing Services (HTS), STI screening and treatment, HIV risk reduction counseling, PrEP, Post Exposure Prophylaxis (PEP), provision of condoms and lubricants, among other services [19]. PrEP retention and adherence information from eligible MSM participants that had attended scheduled routine clinics in the April 2021 to June 2021 quarter. Recruitment of participants and data collection with the aid of a questionnaire happened at the same time for a period of two weeks from 7^th^ September 2021 to 24^th^ September 2021. No incentives were provided; however, participants were reimbursed transport based on public transport fare rates.

### Participants

We enrolled MSM that were at least 18 years old that had been in the KP program for at least 3 months and that had a negative HIV test from their most recent clinical visit. MSM that were receiving ART because of recent seroconversion were excluded from the study.

### Sample size

We originally set out to determine the factors associated with PrEP uptake with a sample size calculation based on using logistic regression for a binary outcome variable on uptake. This was then followed by determining the factors associated with retention on PrEP with a given sample size, based on using Cox regression for a hazard rate ratio, we determined the power. Given that our study also assessed PrEP uptake (not discussed in this paper), the total sample size needed was equal to 751 MSM (448 on PrEP and 303 not on PrEP); with the retention piece focusing on the 448 MSM on PrEP. With a sample size of 448, there was an 80% power at 0.05 significant level to detect a change corresponding to a hazard ratio equal or greater than 2.0.

### Procedures

A list of negative MSM was generated and used to systematically select every nth client until the sample size for the stratum was achieved. In determining the nth sequence, the required sample and PrEP coverage per facility were considered (n = total number of MSM per facility divided by the required sample from the facility), to allow for random distribution of variables under study. The final list of potential study respondents was given to KP peer navigators who conducted community tracing and mobilization of the study participants. A standardized questionnaire was developed and pretested in two health centers providing PrEP but not participating in the study. Findings from the pre-test were used to inform improvements on the tools. All interviews were conducted in either Kinyarwanda or English, depending on the preference of the respondents.

### Measurement of variables

Retention, as a dependent variable in this study was conceptualized as a binary variable. The date of PrEP initiation and that on which the client stopped taking PrEP (interruption) was recorded and retention in weeks calculated as the difference between these two time periods. PrEP interruption was recorded on first instance and no further information was explored in case the same client later reinitiated PrEP. For clients that stayed on PrEP past the 3-month period and were still on PrEP at the time of the study, the interviewer established the date of the 3-month clinic visit and considered it as the stop time for the purpose of calculating 3-month retention. Adherence was assessed through a combination of factors evaluated through factor analysis. Respondents were asked what percent of the time they took all their PrEP medication and an overall response of 95% or more was considered “good adherence” and anything below was considered “poor adherence”. The independent variables in the study were conceptualized as structural, social, clinical, and behavioral factors affecting retention and adherence. Demographic characteristics were also included as independent variables.

### Data handling and statistical analysis

Data collection was conducted by trained study team members using password-protected tablets. Data validation checks were implemented on the data collection software to ensure data consistency and quality. Personally identifiable information (PII) was not collected. Data was relayed daily to a protected local server at Society for Family Health (SFH) offices, which automatically deleted all data on the tablet after synchronization every evening.

Data was cleaned, coded, and uploaded to STATA version 16 for analysis. Descriptive statistics were summarized using percentages. Bivariate analysis was conducted to establish the associations of independent variables with PrEP retention. Independent variables with a *p-value* <0.1 at the bivariate level, were included in the multivariable cox regression proportion hazard model to assess their association with PrEP retention. The factor elimination method was used to reduce the model to the best fit and adjusted odds ratios, 95% Confidence Intervals (95%CIs), and *p*-values were summarized in tables for interpretation.

For PrEP adherence using PCA, we standardized the range of the continuous initial variables so that each one of them contributed equally to the analysis. We computed the co-variance matrices to identify if there were any relationships/correlations between possible pairs of variables and computed the eigen-vectors and eigen-values to determine the principal components of the data. These principal components were considered as new variables constructed as linear combinations or mixtures of the initial variables. These eigen-vectors were then ranked in order of their eigen-values, highest to lowest, to order the principal components by significance. To compute the percentage of variance accounted for by each component, we divided the eigen-value of each component by the sum of eigen-values. This informed whether to keep all components or discard those of lesser significance (of low eigen-values), and to form with the remaining ones a matrix of vectors that we called the ‘Feature-vector.’

### Ethics statement

The study obtained ethical clearance from the Rwanda National Ethics Committee (RNEC) (Ref IRB-00001497) and was reviewed in accordance with the US Centers for Disease Control and Prevention (CDC) human research protection guidelines. Participation in the study was voluntary and formal consent was obtained from participants using a written informed consent form administered in English or Kinyarwanda by one of the study team members and witnessed by another staff member other than the one administering consent. CDC investigators did not interact with human subjects or have access to identifiable data for research purposes. The study investigators ensured that the study achieved anonymity of participants and confidentiality was maintained using study identification (ID) numbers assigned on the questionnaires. Completed questionnaires were electronically stored in password protected devices, and any documents showing the link between ID numbers and target group were kept separately and delinked during analysis.

### Inclusivity in global research

Additional information regarding the ethical, cultural, and scientific considerations specific to inclusivity in global research is included in the (S1 Checklist).

## Results

### Sociodemographic characteristics of respondents

During the study, we interviewed 439 MSM that had enrolled on PrEP from 3 health centers (Biryogo, Gatenga, and Remera) and from the Health Development Initiative (HDI) site. Table 1 shows the socio-demographic characteristics of the MSM respondents reached.

**Table 1:** Sociodemographic characteristic of MSM respondents.

Socio-Demographic Characteristics	Number Enrolled N = 439		Retained on PrEP at 12 weeks		p-value
	N	Col %	N	Row %	
Age-groups					0.974
** 18–24**	139	31.7	124	89.2	
** 25–34**	217	49.4	195	89.9	
** 35–64**	83	18.9	74	89.2	
Self-identify as CSW					<0.001
** No**	255	58.1	214	83.9	
** Yes**	184	41.9	179	97.3	
Currently Employed (Self-reported)					<0.001
** No**	188	42.8	180	95.7	
** Yes**	251	57.2	213	84.9	
*If yes*, *is employment*					
*Formal*	*56*	*22*.*3*			
*Informal*	*195*	*77*.*7*			
Highest Level of Education					0.092
** Primary**	206	46.9	178	86.4	
** Secondary**	210	47.9	195	92.9	
** Post-Secondary**	23	5.2	20	87.0	
Have children					0.686
** No**	335	76.3	301	89.9	
** Yes**	104	23.7	92	88.5	
*If yes*, *how many*?					
** 1**	*43*	*41*.*3*			
** 2**	*43*	*41*.*3*			
** 3+**	*18*	*17*.*4*			
Number of H/H members					0.003
** 1–2**	241	54.9	205	85.1	
** 3–5**	167	38.0	159	95.2	
** 6+**	31	7.1	29	93.5	

The majority of MSM interviewed (n = 217, 49%) were within the age band of 25–34 years followed by the younger ones aged 18–24 years (n = 139, 32%). Older MSM (35–64 years) comprised nineteen percent (n = 83) of the respondents. Forty-two percent (n = 184) of the MSM interviewed self-identified as sex workers (SW), while the rest (n = 255, 58%), said they were non-SW MSM. Of the 439 MSM interviewed, 251 (57%) reported to be employed, while 188 (43%) were not employed. Among the MSM that were employed, most (n = 195, 78%) were employed in the informal sector (doing casual jobs and may not have contractual arrangements and formal guarantees) with only twenty-two percent (n = 56) employed in the formal sector. Forty-seven percent (n = 206) of MSM interviewed reported to have completed primary school education, forty-eight percent (n = 210) completed secondary education while five percent (n = 23) had acquired post-secondary education. The majority of the MSM respondents (n = 335, 76%) didn’t have any children with twenty-four percent (n = 104) having children. Among those MSM those with either one or two children each comprised forty-one percent (n = 43), while seventeen percent (n = 18) had three or more children. Most of the MSM respondents (n = 241, 55%) lived in households of 1–2 persons, followed by those in households of 3–5 members (n = 167), while those that lived in household of 6 or more members comprised seven percent of respondents (n = 31).

### Factors associated with PrEP retention

**Fig 1:**
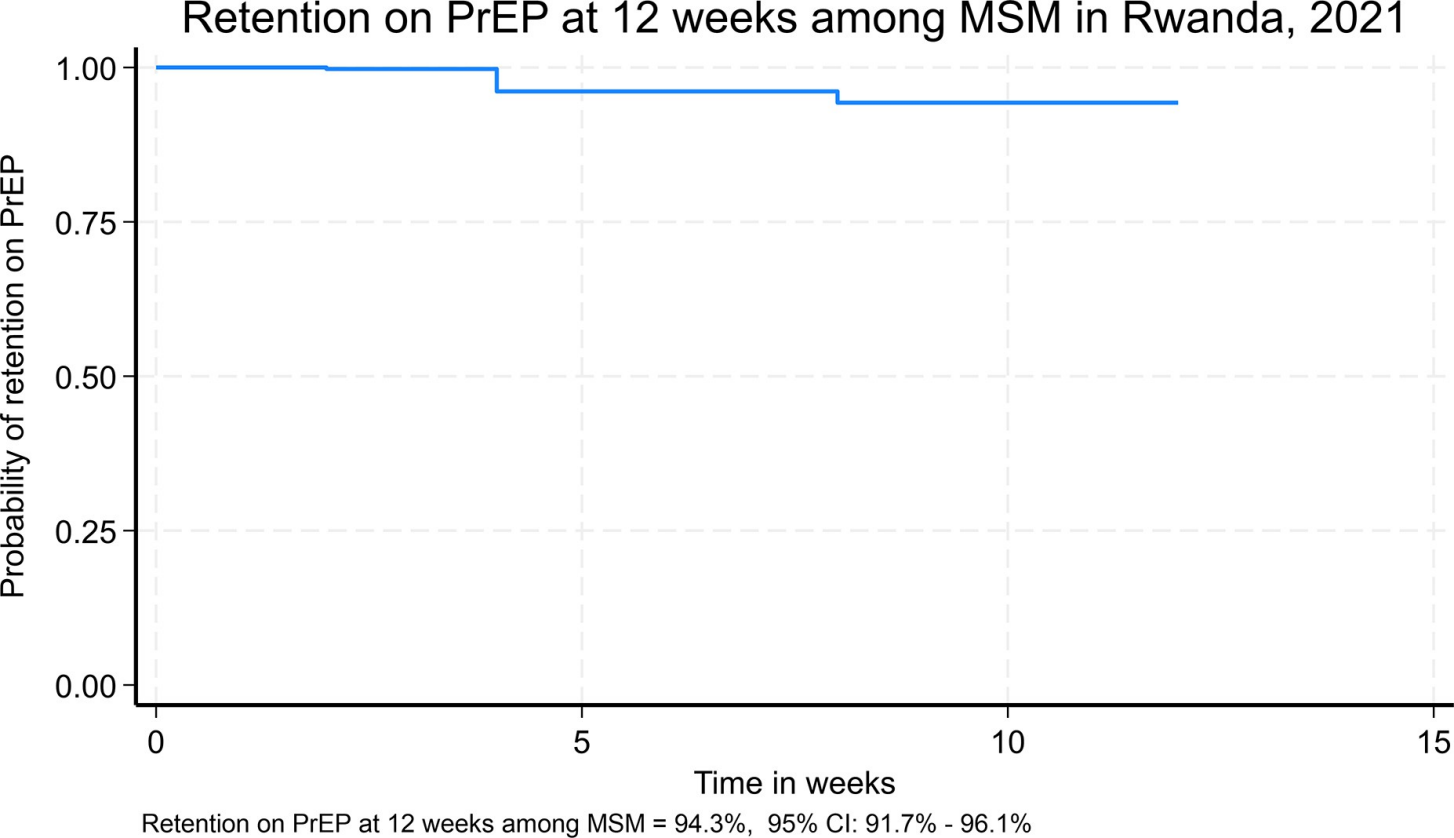
Kaplan-Meier survival probability for MSM initiated on PrEP in Kigali, Rwanda.

Out of the 439 MSM respondents that reported to have taken PrEP (uptake), 393 were still on PrEP by the time of the study, reflecting a PrEP retention rate of 90% (Fig 1).

As shown in Table 2, the multivariable model indicated that MSM who were sex workers were more likely to be retained on PrEP compared to those that were not sex workers (adjusted Hazard Ratio (aHR) = 4.139; 95% Confidence Interval (95%CI): 1.569, 10.921). In addition, MSM that had completed secondary level of education were more likely to be retained on PrEP compared to those that completed only primary level (aHR = 2.154; 95%CI: 1.130, 4.108). Also, MSM living in households of 3–5 members were more likely to be retained on PrEP compared to those living in households of 1–2 members (aHR = 3.755; 95%CI: 1.706, 8.261). MSM that more than one (1) regular sexual partners were more likely to be retained on PrEP compared to MSM with only one (1) regular sexual partner (aHR = 3.949; 95%CI: 2.221, 7.022); and MSM that were circumcised were more likely to be retained on PrEP compared to those that were not circumcised (aHR = 2.218, 95%CI: 1.232, 3.993).

**Table 2:** Frequencies and hazard ratios of PrEP retention associated with covariates among MSM in Kigali.

Section A: MSM					UNADJUSTED MODEL		ADJUSTED MODEL		
Predictor Variable	Ever took PrEP	Retained on PrEP at 12 weeks	FUP Time in weeks	Non-retention Rate /100 person weeks	Crude Hazard Ratio (cHR)	95%CI	Adjusted Hazard Ratio (aHR)	95% CI	P-value
Overall	N = 439	N = 393							
**Age Groups**									
18–24	139	124	5357	0.280	1.000				
25–34	217	195	9075	0.242	1.100	(0.576–2.105)	-	-	-
35–64	83	74	3591	0.251	1.035	(0.456–2.353)	-	-	-
**Commercial Sex worker**									
No	255	214	9,813	0.418	1.000				
Yes	184	179	8,210	0.061	**6.410**	**(2.545–16.129)**	**4.139**	**(1.569–10.921)**	**0.004**
**Employed (self-reported)**									
No	188	180	8,120	0.099	1.000				
Yes	251	213	9,903	0.384	**0.263**	**(0.124–0.558)**	**0.345**	**(0.168–0.707)**	**0.004**
**Highest Level of Education**									
Primary	206	178	7522	0.372	1.000		-	-	-
Secondary	210	195	9442	0.159	**2.104**	**(1.151–3.846)**	**2.154**	**(1.130–4.108)**	**0.020**
Post-Secondary	23	20	1059	0.283	1.239	(0.400–3.840)	0.996	(0.390–2.547)	0.994
**Have Children**									
No	335	301	13617	0.250	1.000				
Yes	104	92	4406	0.272	0.905	(0.470–1.742)	-	-	-
**Number of H/H members**									
1–2	241	205	9675	0.372	1.000				
3–5	167	159	7114	0.112	**3.285**	**(1.527–7.069)**	**3.755**	**(1.706–8.261)**	**0.001**
6+	31	29	1234	0.162	2.307	(0.553–9.636)	1.478	(0.499–4.382)	0.481
Age at 1^st^ Sex Encounter									
<15	21	20	970	0.103	1.000				
15–17	161	140	6637	0.316	0.349	(0.047–2.584)	-	-	-
18–19	124	108	4656	0.344	0.325	(0.043–2.457)	-	-	-
20+	133	125	5760	0.139	0.783	(0.098–6.250)	-	-	-
**Regular sexual partner(s)**									
1	68	47	2357	0.891	1.000				
>1	371	346	15666	0.160	**5.238**	**(2.973–9.226)**	**3.949**	**(2.221–7.022)**	**0.0001**
**Condom use in last 6 months**									
Inconsistent	66	64	2844	0.070	1.000				
Consistent	373	329	15179	0.290	0.249	(0.061–1.061)	-	-	-
**Condom use in last 3 months**									
Inconsistent	84	82	3513	0.057	1.000				
Consistent	355	311	14510	0.303	**0.191**	**(0.047–0.78)**	**0.149**	**(0.035–0.632)**	**0.010**
**Circumcised**									
No	73	60	2840	0.458	1.000				
Yes	366	333	15183	0.217	**2.088**	**(1.105–3.937)**	**2.218**	**(1.232–3.993)**	**0.008**
**PrEP is Highly Stigmatized**									
Not Sure	30	27	1044	0.287	1.000				
Agree	32	27	1406	0.356	0.717	(0.167–3.077)	-	-	-
Disagree	377	339	15573	0.244	1.088	(0.333–3.559)	-	-	-

MSM that were employed had a 66% decreased likelihood to be retained on PrEP compared to those that were not employed (aHR = 0.345; 95%CI: 0.168, 0.707). Also, MSM that used condoms consistently had an 85% decreased likelihood to be retained on PrEP compared to those that did not use condoms for every sexual encounter (aHR = 0.149; 95%CI: 0.035, 0.632).

### Factors associated with PrEP adherence

Most of the MSM respondents (73%, n = 286) ranked their own PrEP adherence as being above 95%. Table 3 includes results from the multivariable principal component regression analysis for MSM. The MSM with principal component involving regular sexual partners had an increased odds of adhering to PrEP as compared to MSM without the principal component involving regular sexual partners (Crude Odds Ratio (cOR) = 1.32; 95% Confidence Interval [95%CI: 1.144, 1.530].

**Table 3:** Output of the PCA regression analysis for MSM.

Principal Component (N = 439)	Unadjusted Odds Ratio	95% CI	P-value
PC 1 (Ever had sex, Age at sex debut, Number of Children)	1.009	0.901, 1.130	0.879
PC 2 (Number of sexual partners, Number of regular sexual partners)	1.323	1.144, 1.530	**<0.001**
PC 3 (Condom use and frequency of condom use in last 3, 6 months)	1.101	0.946, 1.281	0.213
PC 4 (Age, Number residing in household, Status and type of employment)	0.985	0.814, 1.193	0.880
PC 5 (Education status, highest level of education attained)	1.098	0.982, 1.352	0.379

## Discussion

### PrEP retention among MSM

Findings from this study revealed that MSM that were commercial sex workers, attained secondary as highest level of education, lived in a household of 3–5 members, had more than one sexual partner, and were circumcised, had higher odds of being retained on PrEP at 3-months while those that were employed and those that reported consistent condoms 3-month prior to the interview had lower odds of being retained on PrEP at 3-months.

Our study assumes that perceived risk to HIV acquisition, as would be the case with sex worker MSM, would be a motivation to PrEP retention. This finding is similar to other published studies where perceived HIV risk emanating from sexual activity influenced both PrEP uptake and PrEP retention [20, 21]. Sex work exposes those involved with multiple sexual partners and in circumstances where one is not able to use condoms, PrEP retention remains an effective measure against HIV acquisition [22]. It thus follows that MSM who are supported to properly assess their risk of HIV acquisition are more likely to be retained on PrEP, should that risk persist.

Our study demonstrated that MSM that had completed secondary education were more likely to be retained on PrEP. This study is consistent with other studies that show higher levels of education being associated with good retention on PrEP [23–25]. Those with better education have been demonstrated to have greater access to information, better understanding of HIV and ultimately a better appraisal of their HIV risk hence reinforcing retention on PrEP [26, 27]. Thus, PrEP retention efforts need to have special consideration for those MSM with no or lower educational attainment.

This study showed that living in a household of 3–5 people was associated with increased odds of being retained on PrEP. While high household number have been demonstrated to have negative effects on retention modulated through stigma, this finding is plausible if the members of the household are supportive and have been disclosed to or the other household members themselves are using PrEP [28, 29].

There is limited literature linking uptake of voluntary male medical circumcision (VMMC) with PrEP retention. In this study, we propose that individuals (including MSM) who are at heightened risk of HIV acquisition, and whose HIV risk perception is concordant with this increased risk, are more likely to take on multiple risk reduction strategies, in this case VMMC and PrEP. There is growing evidence to show that interventions to improve retention on PrEP are also good at averting new HIV infections. Being fully adherent to PrEP is the most effective strategy in reduction of HIV acquisition, but given the issues that can hamper perfect adherence, a combination strategy (VMMC inclusive) is likely to increase the HIV protective effect, compared to interventions that focus on improving adherence alone [30]. It follows that MSM retained on PrEP are likely to be supported with adherence issues while those not retained will face challenges regarding adherence to PrEP [31].

Our study revealed that employed MSM were less likely to be retained on PrEP. This finding is similar to what was seen in Chicago from the RADAR study—the longest running longitudinal cohort study of Lesbian Gay Bisexual and Transgender (LGBT) youth ever conducted [32]. In their study among MSM in West Africa, Eubanks et al, established findings to the contrary with unemployed MSM having increased odds of being lost to follow-up compared to their employed counter-parts [33]. Nevertheless, keeping a PrEP schedules can be a barrier to PrEP retention and its thus plausible that busy schedules at work might hinder the ability of working MSM from honoring scheduled appointments [34].

Fears have been raised about behavioral inhibition with some studies reporting increase of condomless sex among clients enrolled on PrEP [35–37]. Some studies have shown the opposite effect where PrEP was associated with decrease in condomless sex: results being attributable to additional education when PrEP was offered as part of a combination prevention strategy [38, 39]. Other studies have demonstrated a neutral position with no change in sexual behavior [40–42]. One study in the US reported increase in condomless sex during periods of non-PrEP use which was a worrying finding [43]. Our study does not provide any evidence for or against behavioral inhibition. However, it showed low PrEP retention among consistent condom users suggesting non-adoption of a combination prevention strategy thus presenting an opportunity for improvement.

### PrEP adherence among MSM

In this study, we found that MSM with a principal component involving regular sexual partners had higher odds of adhering to PrEP as compared to MSM without a principal component involving regular sexual partners. The choice to have stronger adherence on PrEP in situations of high risk exposure, as would be the case with an MSM with multiple sexual partners, has been demonstrated elsewhere [44–46]. The explanation behind this leans more towards self-preservation which is a psychological construct denoting that the need to preserve oneself underlines social behavior [47]. We postulate that if MSM were supported in evaluating their perceived and actual HIV risk more accurately, we would observe better PrEP adherence measures.

As a study strength, the retrospective cross-sectional design allowed for multiple phenomena to be explored within a limited span of time, allowing early insights into the PrEP program in Kigali, and providing data to guide the national expansion of the PrEP program among MSM. Our study faced several limitations: The retrospective cross-sectional study design made it difficult to determine the temporality of the observed findings, thus limiting our ability to succinctly build a case for cause-effect relationships. Several responses in our study were self-reported with a potential for response bias. Checks were built into the questionnaire and interviewers were trained to establish rapport with the respondents to create an atmosphere where respondents are free and likely to answer questions correctly. The study was also limited in its ability to establish whether clients who were reported as currently not on PrEP had ever initiated or reinitiated PrEP, and if so, how many times. This would have allowed incorporation of the fact that the decision to continue or discontinue PrEP is usually dependent on the client’s perceived risk, which is never a static phenomenon. In some settings, this is being addressed by event driven PrEP (ED PrEP) where clients only take PrEP during times of anticipated risk of exposure; long-acting PrEP provided as injectable, or a vaginal ring. Our study relied on self-reported adherence, and this could have potentially under-estimating true adherence. While we used a high cut-off of 95% or more to minimize this reporting bias, a residual bias is likely to persist and thus blood measures would have been more accurate.

Future studies could creatively consider using blood measures in determining PrEP adherence although those are currently challenging to achieve within the financial limits of KP and PrEP programming.

## Conclusions

Our study provides insights into PrEP retention and adherence among MSM in Rwanda. We established that MSM who were sex workers, lived in a household of 3–5 members, and those that had more than one more regular sexual partners, completed secondary education, and were circumcised had higher odds of PrEP retention. On the other hand, MSM who were employed and those who used condoms consistently were less likely to be retained on PrEP. Our findings demonstrate that MSM that had higher numbers of sexual partners, presented with increased odds of adhering to PrEP.

The findings can guide HIV prevention efforts in a way that MSM who engage in risky sexual behavior should be encouraged to add PrEP to other HIV prevention strategies at their disposal to increase their level of protection. Additionally, Community PrEP refills can also close PrEP adherence and retention gaps observed among employed MSM who find it challenging to honor their scheduled appointments. However, in Rwanda, community PrEP refill is not yet policy and advocacy for its adoption remains an on-going campaign for which this study adds evidence in support of the same. KP programs should continue to emphasize PrEP use alongside other known HIV prevention strategies, and targeted STI testing, and treatment prioritized for those that might not use condoms alongside PrEP.

## Supporting information

S1 Checklist Inclusivity in global research. (DOCX)
